# Identification of novel cell-impermeant fluorescent substrates for testing the function and drug interaction of Organic Anion-Transporting Polypeptides, OATP1B1/1B3 and 2B1

**DOI:** 10.1038/s41598-018-20815-1

**Published:** 2018-02-08

**Authors:** Izabel Patik, Virág Székely, Orsolya Német, Áron Szepesi, Nóra Kucsma, György Várady, Gergely Szakács, Éva Bakos, Csilla Özvegy-Laczka

**Affiliations:** 1 Membrane protein research group, Institute of Enzymology, Research Centre for Natural Sciences, Hungarian Academy of Sciences, Magyar tudósok krt. 2, Budapest, H-1117 Hungary; 2 Laboratory of Molecular Cell Biology, Institute of Enzymology, Research Centre for Natural Sciences, Hungarian Academy of Sciences, Magyar tudósok krt. 2, Budapest, H-1117 Hungary; 3 Institute of Cancer Research, Medical University of Vienna, Borschkegasse 8A, Vienna, 1090 Austria

## Abstract

Organic Anion-Transporting Polypeptides are multispecific membrane proteins that regulate the passage of crucial endobiotics and drugs across pharmacological barriers. OATP1B1 and OATP1B3 have been described to play a major role in the hepatic uptake of statins, antivirals and various chemotherapeutics; whereas the pharmacological role of the ubiquitously expressed OATP2B1 is less well characterized. According to current industry standards, *in vitro* testing for susceptibility to OATP1B1 and 1B3 mediated transport is recommended for drug candidates that are eliminated in part via the liver. Here we show that human OATP1B1, 1B3 and 2B1 transport a series of commercially available viability dyes that are generally believed to be impermeable to intact cells. We demonstrate that the intracellular accumulation of Zombie Violet, Live/Dead Green, Cascade Blue and Alexa Fluor 405 is specifically increased by OATPs. Inhibition of Cascade Blue or Alexa Fluor 405 uptake by known OATP substrates/inhibitors yielded IC_50_ values in agreement with gold-standard radioligand assays. The fluorescence-based assays described in this study provide a new tool for testing OATP1B/2B1 drug interactions.

## Introduction

Human Organic Anion-Transporting Polypeptides (OATPs) encoded by the SLCO genes mediate the cellular uptake of large organic, amphipathic molecules^1,2^. At least four members of the family, OATP1A2, 1B1, 1B3 and 2B1 are multispecific transporters that, besides the transport of endogenous substrates (bilirubin, bile acids and hormones), also promote the cellular uptake of pharmacologically relevant molecules. OATP1B1 and 1B3 are almost exclusively expressed in the sinusoidal membranes of hepatocytes where they regulate the hepatic uptake of bile acids and bilirubin. Simultaneous mutations in the SLCO1B1 and 1B3 genes result in Rotor syndrome, characterized by increased serum bilirubin levels^3^. Additionally, OATP1B1 and 1B3 are key determinants of the hepatic clearance of widely prescribed medications (e.g. statins, antivirals) and also of chemotherapeutics including docetaxel, irinotecan and cisplatin^4,5^. Altered function of OATP1B1 and 1B3 due to single nucleotide polymorphisms (SNPs), drug-drug or drug-food interactions or disease conditions influences the *in vivo* efficacy of drugs^6,7^. Co-administration of OATP1B substrate drugs may cause unexpected toxicity with fatal consequences. For example, statin-induced myopathy was shown to be linked to the inhibition of transporter-mediated hepatic uptake of statins by the co-administered gemfibrozil or Cyclosporin A^8,9^. Inhibition of OATP1B function may also result in elevated bilirubin levels^10,11^. OATP1B expression is often reduced in liver diseases including non-alcoholic fatty liver disease, hepatocellular carcinoma, inflammatory cholestasis, primary biliary cirrhosis or chronic hepatitis^12^. OATP2B1 is also expressed in the liver^13^, though its contribution to the hepatic clearance of exogenous compounds is unclear. OATP2B1 was shown to influence the intestinal absorption of orally administered drugs such as celiprolol, fexofenadine and montelukast^5,14^. Additionally, OATP2B1 is expressed in skeletal muscle and in the heart, mediating the muscular uptake and myotoxicity of statins^15^. OATP1A2, the fourth multispecific member of the OATP family, has a largely overlapping expression pattern with OATP2B1, e.g. in the intestine and the blood-brain-barrier^5,16^. Additionally, OATP1A2 is present in the liver, however in contrast to OATP1Bs and 2B1, 1A2 is found in cholangiocytes^17^. Therefore, although OATP1A2 transports a plethora of clinically applied drugs, it is not directly involved in hepatic drug uptake, but rather in the reabsorption of drugs from the bile. Based on pre-clinical and clinical data, OATP2B1 and 1A2 are key determinants of the intestinal uptake of numerous drugs, including various statins, fexofenadine, sulfasalazine and telmisartan^18^.

Recent guidelines issued by the US Food and Drug Administration (FDA) and the European Medicines Agency (EMA) require testing the interaction of new molecular entities with OATP1B1 and 1B3^19,20^, and OATP2B1 and OATP1A2 are emerging candidates according to the International Transporter Consortium^20^. Recommended functional assays typically measure the effect of the investigated compounds on the OATP-mediated uptake of radioactively labelled compounds^7^. Typical test substrates of OATP1B and 2B1 include radioactively labelled estrone-3-sulphate, estradiol-glucuronide, bromosulphophthalein, a statin or cholecystokinin-8 (1B3)^7^. Recently, several clinically applied drug substrates of OATP1B1 (various statins, fexofenadine, or bosentan) measured by HPLC-MS (high-performance liquid chromatography with tandem mass spectrometry) have been shown to be applicable as test substrates to predict DDI^21^. Whereas these indirect assays provide a reliable and sensitive measurement of OATP function, radioactive compounds and MS are usually not compatible with large scale screening efforts. Lately, ^3^H-Rosuvastatin and DHEAS have been demonstrated as *in vivo* substrates of OATP1Bs in cynomolgus monkey^22,23^, and erlotinib as a potential probe substrate for OATP2B1 applicable in humans^24^.

Fluorescence-based detection technologies are frequently applied in biological testing, due to their unique advantages in setting up homogeneous, sensitive assays in miniaturized formats^25^. A common feature of drug transporters is their wide substrate specificity that also encompasses fluorescent molecules. Indeed, fluorescent molecules have been successfully used in *in vitro* and *in vivo* transporter assays^26^. Calcein-AM, originally developed as a viability dye, was discovered to be a high affinity substrate of several pharmacologically relevant ABC transporters^27–29^. Similarly, Hoechst 33342 and DyeCycle Violet, two nucleotide/DNA binding dyes, are ABCG2 and ABCB1 substrates that can be used to characterize transporter function^30,31^. Screening assays based on the OATP1B1/3-mediated uptake of fluorescein, fluorescein-methotrexate or various fluorescein derivatives have also been developed^32–34^. The applicability of fluorophores in transporter-based assays depends on several sources of potential artefacts, including non-specific protein binding, sequestration within the cell, or quenching by pH or intracellular ions. Unlike fluorescein or fluorescein-methotrexate, an ideal OATP test substrate should be membrane impermeable, and its fluorescence should be independent of the intracellular milieu.

Our aim in this study was to expand the scope of currently available fluorescent indicators of hepatic OATPs, 1B1, 1B3 and 2B1. In particular, we wanted to identify a pH-independent fluorophore with low cell permeability, to ensure a high signal to noise ratio and to allow transport measurements at acidic pH levels needed for the optimal activity of OATPs^35^. Using cell lines engineered to overexpress human OATP1B1, 1B3, or 2B1, we identify a series of commercially available cell impermeable dyes as high affinity OATP substrates. We show that a transport assay based on the uptake of the best-performing fluorophores is amenable to semi high-throughput screening for OATP drug interactions.

## Results

### Zombie Violet is a novel substrate of human OATPs, 1B1, 1B3 and 2B1

**Figure 1:**
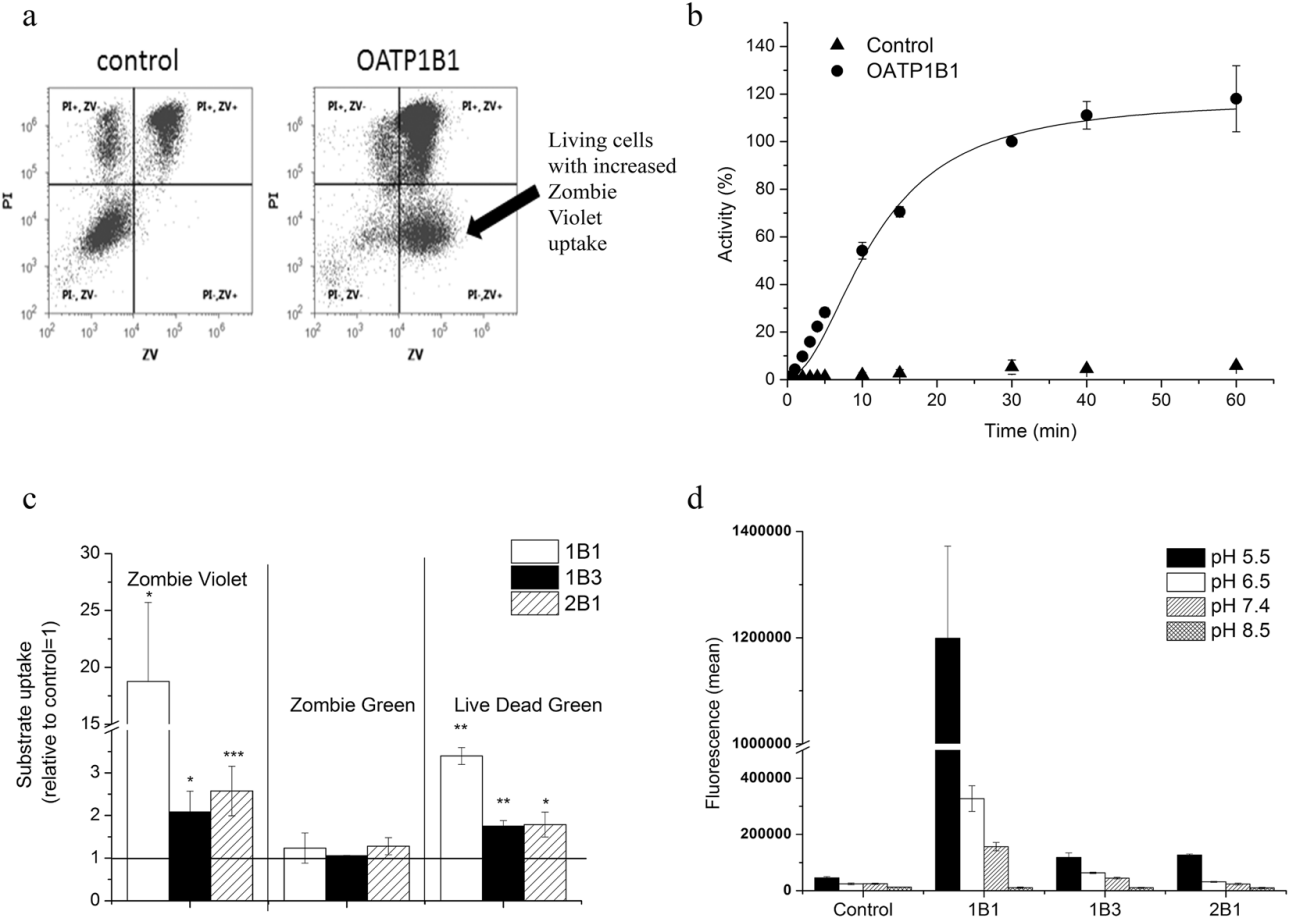
Uptake of viability dyes in Sf9 cells measured by flow cytometry. (**a**) Uptake of ZV (0.2 µl in 100 µl) was measured at 37 °C in pH 5.5 uptake buffer for 15 minutes. Dead cells were identified based on PI staining. Experiments were repeated at least three times, the result of one representative experiment is shown. (**b**) Kinetics of OATP1B1-mediated ZV uptake. Uptake rates were normalized to the fluorescence values measured for OATP1B1 incubated with 2 µl ZV for 30 minutes. (**c**) ZV and LDG uptake in Sf9 cells. Dye (0.2 µl in 100 µl) uptake was measured after 30 minutes of incubation. Statistical analysis was performed by Student’s t-test. *p < 0.05, ***p < 0.001. (**d**) pH dependent uptake of ZV in Sf9 cells. Uptake of 0.2 µl ZV in 100 µl at 37 °C was measured in buffers with different pH for 10 minutes. (**b**,**c** and **d**) data represent the average of three independent experiments ± SD values.

In an effort to identify new fluorescent substrate candidates of hepatic OATPs, we searched the literature for dyes showing low membrane permeability and pH independent fluorescence. Based on these characteristics we focused on commercially available viability dyes, because these fluorescent molecules do not stain living cells, and therefore are expected to show low passive permeability. Recently, we have shown that the OATP-mediated transport of fluorescent compounds can be quantitatively monitored in insect cells^36^. Therefore, first we used this expression system to test the contribution of OATP1B1 to the cellular uptake of Zombie Violet™ (ZV, BioLegend), an amine-reactive fluorescent dye used for the labelling of dead cells. To discern staining due to cell death, we counterstained the cells with propidium iodide (PI). Whereas in control cells staining with ZV was always accompanied by PI-positivity (indicating a loss of membrane integrity), cells expressing the human OATP1B1 transporter were distinctly ZV-positive and PI-negative, indicating that ZV cannot penetrate living cells unless OATP1B1 is present in the plasma membrane (Fig. 1a). Detailed transport measurements confirmed that the accumulation of ZV is due to OATP1B1-mediated uptake, showing saturable (Fig. 1b) and inhibitor-sensitive uptake (Supplementary Figure 1a).

Next, we screened ZV against the other, multispecific human OATPs of the liver, 1B3 and 2B1 (Fig. 1c). We found that, albeit to a lesser extent, ZV is also transported by OATP1B3 and 2B1. ZV transport was sensitive to pH (Fig. 1d), with highest uptake at pH 5.5; and also to inhibitors (Supplementary Figure 1b), indicating that ZV is a bona fide OATP1B and 2B1 substrate. Additionally, we found that Live/Dead Green (LDG, Life Technologies), also designed to label dead cells, is another substrate of these three OATPs, while in the case of Zombie Green (a viability dye from the Zombie^TM^ family), there was no OATP-mediated transport (Fig. 1c).

### Establishment of A431 cells with robust OATP1B1, 1B3 or 2B1 expression using viability dye-based cell sorting

**Figure 2:**
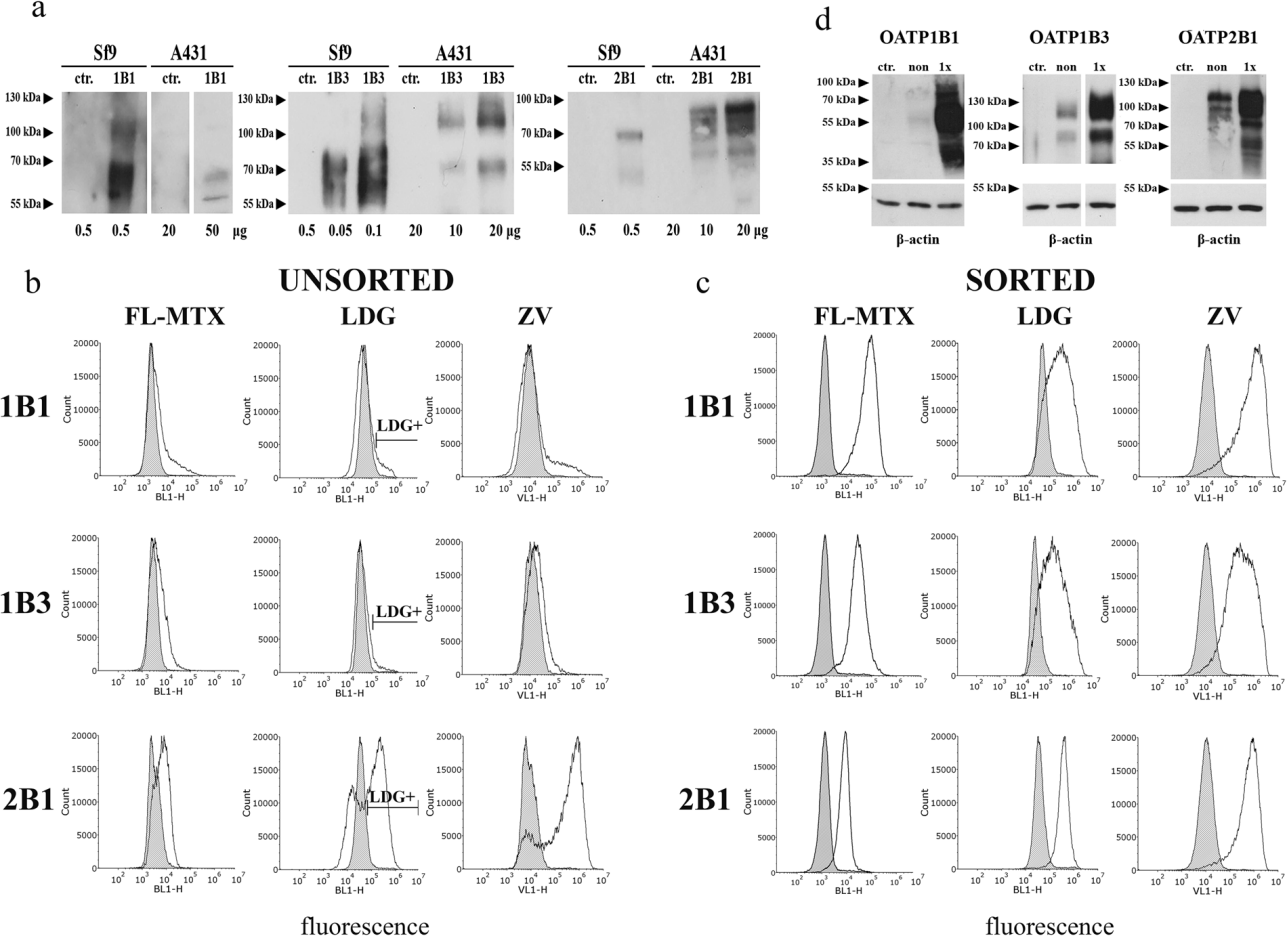
Low level of OATP1B expression in A431 cells. (**a**) Western blot detection of human OATPs expressed in insect and A431 cells. Total cell lysates were analysed by Western blot. Control (ctr.) represents Sf9 cells expressing an unrelated protein or mock transfected A431 cells. Multiple migratory bands most probably represent differently glycosylated forms of OATPs. Figure for OATP1B1 was sliced from the same blot, same exposure time. Full-length blots are presented in Supplementary Figure 7. (**b**) Fl-MTX, LDG and ZV uptake in A431 cells. Representative histograms show the uptake of 1 µM Fl-MTX or 0.4 µl LDG or ZV into A431 cells before and after sorting. Cells with the highest LDG fluorescence were sorted, and after recovery, the cells were again measured for LDG uptake (panel c). Mock transfected control cells are indicated by filled histograms. Cells were incubated with the substrates for 15 minutes (Fl-MTX) or 30 minutes (ZV, LDG) at 37 °C in uptake buffer (pH 5.5) in final volume of 100 µl. Living (PI-negative) cells are shown. (**d**) LDG sorting results in increased expression of OATP1B1, 1B3 and 2B1. OATP expression was determined using whole cell lysates (20 μg each) by Western blot. A431 mock transfected cell lysates were used as control. β-actin served as an internal control. Experiments were repeated at least twice. One representative blot is shown. Ctr.: mock-transfected, non: non-sorted, sort: sorted. Multiple migratory bands most probably represent differentially glycosylated forms of OATPs. Full-length blots with different exposition times are presented in Supplementary Figure 7.

While the Sf9 system has several advantages, the transient nature of OATP expression is not compatible with high-throughput screening (HTS). In order to build a stable model system and to test whether OATP1B and 2B1-mediated viability dye uptake can also be observed in human cells, we generated A431 (human epidermoid carcinoma) cell lines overexpressing OATP1B1, 1B3 or 2B1. The A431 cell line was chosen based on its good adherence necessary for transport measurements in 96 well plates. OATP2B1 was readily overexpressed in A431 cells, whereas expression levels of OATP1B1 and 1B3 remained very low despite repeated rounds of puromycin selection or lentiviral transduction (Fig. 2a shows OATP protein levels in A431 cells in comparison to the levels achieved in insect cells). Consequently, transport of a common OATP1B and 2B1 substrate, fluorescein-methotrexate showed weak OATP1B activity compared to OATP2B1 (Fig. 2b). Similarly, convincing ZV and LDG uptake could only be observed in A431-OATP2B1 cells (Fig. 2b).

Substrate uptake is proportional to OATP expression/function, and we sought to determine whether subpopulations with increased OATP expression could be identified based on substrate accumulation. Since LDG is well tolerated (see Supplementary Figure 2), we sorted highly fluorescent LDG-positive A431-OATP1B1, 1B3 and 2B1 cells, which were further propagated in cell culture. Stunningly, the sorted cells showed significant OATP expression and function (Fig. 2c,d), indicating that preferential uptake of LDG allowed the function-based sorting of cells with high OATP expression. High expression levels were maintained for at least 2 months (cca. 20 passages) without the need of further sorting or selection.

### A set of commercially available fluorophores as OATP1B and 2B1 substrates

**Figure 3:**
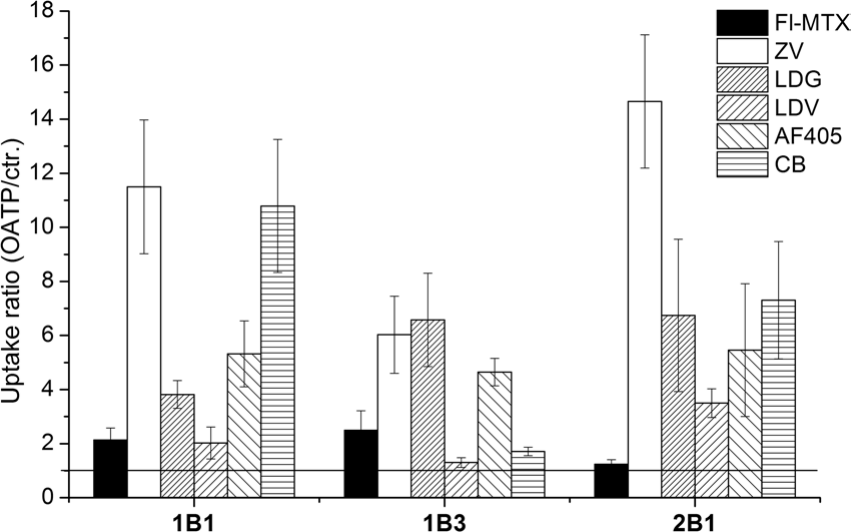
Screening identifies additional fluorescent OATP substrates. Transport was determined in A431 cells expressing OATP1B1, 1B3 or 2B1 seeded in 96-well plates. The cells were incubated with 1 µl ZV, LDV, LDG, 1 µM (or 4 µM for OATP2B1) Fl-MTX or 20 µM CB and AF405 for 30 minutes at 37 °C in buffer with pH 5.5, in final reaction volume of 100 µl. Fluorescence was determined using an Enspire fluorescent plate reader. Activity was calculated by dividing fluorescence measured in A431-OATP cells with that measured in A431 mock transfected cells. Average of at least three independent measurements with triplicates ± SD values are shown.

In addition to ZV and LDG, a large panel of spectrally diverse dyes aimed for the detection of dead cells is available commercially (Table 1). In order to find out whether these fluorescent dyes are also recognized by OATP1B1, 1B3 and 2B1, we monitored their uptake in 96 well plates using the sorted A431 cells. In addition to the viability dyes, we tested the transport of two other cell-impermeant fluorescent compounds, Cascade Blue hydrazide (CB) and Alexa Fluor 405 succinimidyl ester (AF405), intended for use in cell permeability assays and the fluorescent labelling of proteins, respectively. As shown in Fig. 3, we found a robust fluorescent signal in OATP-expressing cells with several dyes. Moreover, in the case of ZV, LDG, CB and AF405 the signal intensity highly exceeded that of Fl-MTX, indicating that the newly identified dyes may be better suited for fluorescence-based studies assaying OATP function. On the other hand, Zombie Green, Live/Dead Red, Live/Dead Aqua, Live/Dead Yellow, Live/Dead Far-red and Live/Dead near-IR were not transported by the investigated OATPs (Table 1).

**Table 1:** List of the fluorescent dyes tested in the current study.

Distributor	Dye	Ex/Em optimum (nm)	Transported by OATPs in A431 cells
BioLegend	Zombie Green	488/515	—
	Zombie Violet	405/423	1B1, 1B3, 2B1
Thermo Fisher (Life Technologies)	Live/Dead Blue	350/450	very weak 1B1, 1B3, 2B1
	Live/Dead Aqua	367/526	—
	Live/Dead Violet	416/451	1B1, 1B3, 2B1
	Live/Dead Yellow	400/575	—
	Live/Dead Green	495/520	1B1, 1B3, 2B1
	Live/Dead Red	595/615	—
	Live/Dead Far-red	650/665	—
	Live/Dead near-IR	750/775	—
	Alexa Fluor 405 NHS Ester	401/421	1B1, 1B3, 2B1
	Cascade Blue Hydrazide	400/419	1B1, 1B3, 2B1

All dyes were tested for transport by OATP1B1, 1B3 and 2B1 expressed in A431 cells in 96-well plates using an Enspire fluorescent plate reader. Transport was tested in at least two independent experiments using triplicates. “—”indicates lack of OATP-mediated uptake.

### Functional assay adapted to microplates

In order to find optimal conditions for measuring the uptake of the newly identified OATP1B and 2B1 substrates and to choose the best dye that could be applied in a semi high-throughput set up, we characterized the kinetics of uptake by A431-OATP1B1, 1B3 and 2B1 cells seeded in 96-well plates. We found rapid (t_1/2_ values around 10–15 minutes, Supplementary Figure 3b) uptake of the newly identified fluorescent dye substrates, and most importantly we also observed that incubation/reaction time could be prolonged up to 60 minutes without significant “leakage” of the dyes into control cells. The optimum condition for OATP1B and 2B1-mediated uptake for all the tested dyes was found to be at pH 5.5 (Fig. 1d, and Supplementary Figure 4).

**Figure 4:**
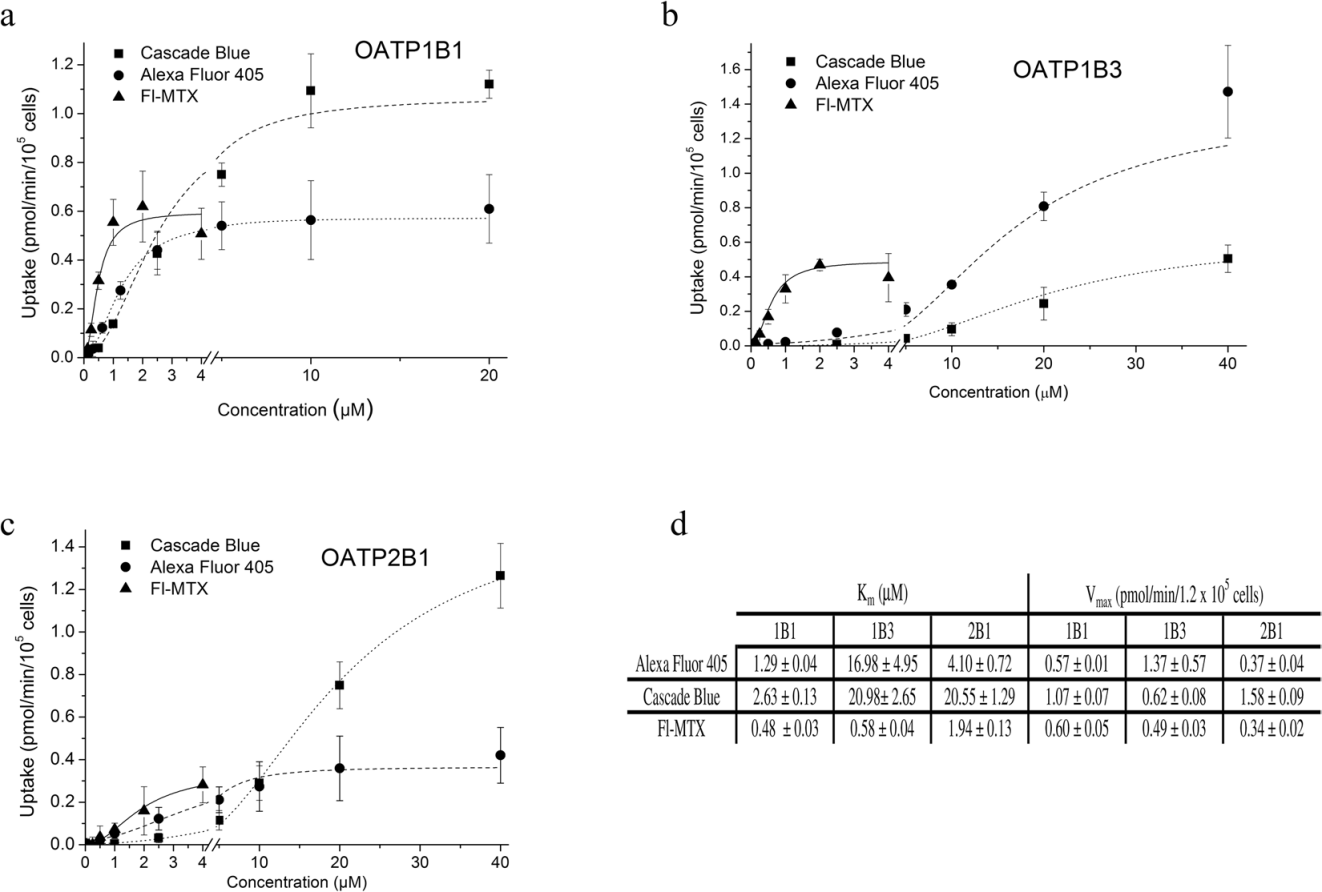
Kinetics of uptake of CB, AF405 and Fl-MTX in A431 cells overexpressing OATP1B1, 1B3 or 2B1. Transport was measured in 96-well plates. Cells were incubated with increasing concentrations of Fl-MTX, CB or AF405 in the linear phase of uptake (2.5 minutes for Fl-MTX, 10 minutes (1B1, 2B1) or 15 minutes (1B3) for CB, and 15 minutes for AF405). Transport capacity was calculated based on calibration with known amounts of the dye. Uptake in A431-OATP cells without background signal (fluorescence in A431-mock cells) is shown. Data points indicate average ± SD values obtained in three independent experiments.

Structural information was available for AF405 (Tris(N,N-diethylethanaminium) 8-[2-(4-{[(2,5-dioxopyrrolidin-1-yl)oxy]carbonyl}piperidin-1-yl)-2-oxoethoxy]pyrene-1,3,6-trisulfonate) and CB ([(3,6,8-trisulfo-1-pyrenyl)oxy]-,1-hydrazide), which were further characterized to determine the kinetic parameters of transport (see Supplementary Figure 3 for detailed characterization of ZV, LDV and LDG transport). In comparison to Fl-MTX, AF405 and CB proved to be lower affinity substrates, whereas the V_max_ of 1B1/2B1-mediated CB transport was 2–4 fold higher. 1B3 showed weak CB transport, but AF405 proved to be an excellent substrate with cca. 3-fold higher V_max_ value as compared to Fl-MTX (Fig. 4). In experiments performed at ideal conditions for each dye, we found that the maximum signal (OATP vs. vector control) can be achieved with ZV for all three OATPs. CB is as good a substrate for OATP1B1 and 2B1 as ZV. In the case of OATP1B3 the highest signal was achieved with ZV, LDG and AF405 (activity ratios are summarized in Table 2). Importantly, the transport of all novel fluorescent dyes could be inhibited by known inhibitors.

**Table 2:** Transport ratio and z-factor determined in A431-OATP cells.

	Transport ratio compared to control			z-factor		
	1B1	1B3	2B1	1B1	1B3	2B1
Fl-MTX	2.14 ± 0.44	2.49 ± 0.72	1.24 ± 0.16	**0.59**	**0.77**	−1.80
**Zombie Violet**	**11.05 ± 2.47**	**6.03 ± 1.43**	**14.65 ± 2.47**	**0.61**	**0.61**	**0.71**
Live/Dead Violet	2.03 ± 0.59	1.33 ± 0.21	3.50 ± 0.53	**0.55**	−0.42	**0.79**
**Live/Dead Green**	**3.82 ± 0.51**	**6.58 ± 1.73**	**6.74 ± 2.82**	**0.84**	**0.76**	**0.62**
**Cascade Blue**	**10.79 ± 2.46**	1.71 ± 0.16	**7.31 ± 2.17**	**0.73**	0.26	**0.66**
**Alexa Fluor 405**	**5.32 ± 1.22**	**4.65 ± 0.51**	**5.46 ± 2.46**	**0.59**	**0.64**	**0.57**

A431 cells (seeded in 96-well plates) were incubated with the dyes at pH 5.5 for 30 minutes in order to reach maximum fluorescence signal. Data were calculated from at least 3 independent measurements. Dyes were applied in the following concentrations/amounts: Fl-MTX 1 µM (1B1 and 1B3) and 4 µM (2B1); ZV, LDV and LDG 1-1 µl; CB and AF405 10 µM (1B1 and 2B1) and 20 µM (1B3). A z-factor above 0.5 is defined as an excellent assay^37^. Dyes defined as best candidates for HTS are indicated in bold.

### Inhibition assay using Cascade Blue or Alexa Fluor 405 to probe substrates

**Figure 5:**
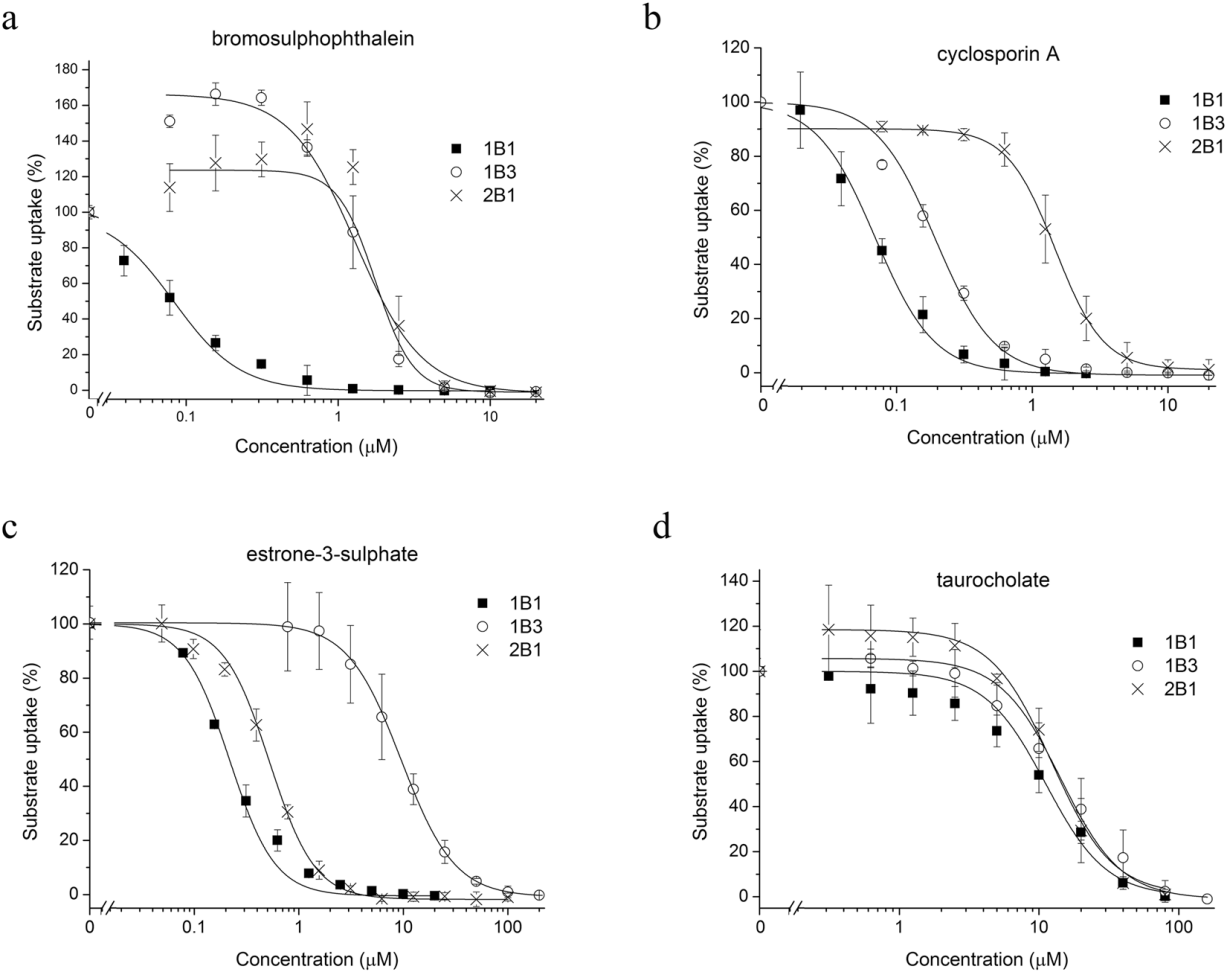
Inhibition of CB and AF405 uptake in A431-OATP cells. Transport of CB (2 µM for OATP1B1 and 10 µM for OATP2B1) and AF405 (5 µM, OATP1B3) was measured for 30 minutes in the absence or presence of the investigated compounds. Transport was determined by subtracting fluorescence in A431-mock cells. Transport measured in cells with the dye alone was set to 100% and the effect of the compounds was compared to this value. Experiments were performed in triplicates with three parallels in each biological replicate. Average ± SD values are shown.

Next, we tested the applicability of the best performing substrates, CB for OATP1B1 and 2B1, and AF405 for OATP1B3 to detect OATP drug interactions. We measured the inhibitory effect of four well-known OATP1B and 2B1 interacting compounds (Cyclosporin A (CsA), bromosulphophthalein (BSP), taurocholate (TC) and estrone-3-sulphate (ES)). All four compounds inhibited CB or AF405 uptake in a concentration dependent manner (Fig. 5). Moreover, the IC_50_ values obtained in the fluorescence-based assays (Table 3) showed perfect agreement with results obtained using radioactive substrates, and pilot screens yielded a z-factor above 0.5, suggesting that the new, fluorescence-based assays are amenable to HTS detecting OATP1B/2B1 drug interactions (Table 2)^37^.

**Table 3:** Comparison of IC_50_ values with literature data.

	OATP1B1		OATP1B3		OATP2B1	
	This study	Literature data	This study	Literature data	This study	Literature data
BSP	0.08 +/− 0.10	0.1^34,41,45^	0.9+/−0.31	0.4^55^, 0.5^41^	1.26+/− 0.56	1.2^46^
CsA	0.07 +/− 0.04	0.1^34,45^	0.18+/−0.05	0.2^41^, 1.2^44^	1.45+/− 0.11	36^44^
		0.2^41^, 1.3^44^				
E1S	0.22 +/− 0.004	0.05^34,41^	9.5+/−0.13	20^41^	0.56+/− 0.05	
TC	11.2 +/− 0.22	9^34^, 19^45^	14.3+/−0.32	18*^56^	12.3+/− 0.15	9^46^

IC_50_ (µM) values were determined using 2 or 10 µM CB (OATP1B1 or 2B1) or 5 µM AF405 (OATP1B3). For detailed description see Fig. 5. Literature data show IC_50_ values obtained in assays using EG, ES, 8-FcA or DCF as probe substrates. When transport inhibition data were not available, Km values obtained in direct transport experiments are shown, indicated by *.

## Discussion

Testing the interaction between a new molecular entity and OATP1Bs is required at early stages of drug development. Several fluorescent OATP1B substrates have already been identified. These substrates are either molecules with intrinsic fluorescence, mainly fluorescein and its derivatives^33,34^, or OATP substrates tagged with a fluorophore, such as Oregon green/Flutax-2 (1B3^32^), chenodeoxycholyl-(Nε-1 nitrobenz-2-oxa-1,3-2 diazole)-lysine (CDCA-NBD)^38^, cholyl-glycylamido-fluorescein (CGamF)^39^, cholyl-L-lysyl fluorescein (CLF)^40^, fluorescein-methotrexate (Fl-MTX)^32^ and 8-fluorescein-cAMP (8-FcA)^41^. Whereas methods based on the uptake of fluorescein and fluorescein-methotrexate have been adapted to semi high-throughput (HT) format^32,33^, due to reliability, sensitivity and availability issues, most of these probes are not ideal for large scale OATP drug interaction screens^34^. For example, Gui *et al*. found that due to its lower transport capacity by OATP1B1, Fl-MTX is not suitable for HT OATP1B1 drug interaction screening^32^. Moreover, in the case of OATP2B1, an emerging candidate in pharmacokinetic studies, no such fluorescence-based large scale screening method has been reported.

Here our aim was to identify novel fluorescent OATP1B1, 1B3 and 2B1 substrates. We tested commercially available fluorescent molecules showing low passive cellular uptake, high fluorescence quantum yield and pH insensitivity. These characteristics are required to achieve high signal to noise ratio, and maximal OATP activity, since OATP2B1 functions (almost) exclusively at acidic extracellular pH^36,42^. Based on these criteria, we chose two sets of compounds. First, we tested fluorescent viability dyes (Zombie dyes and the Live/Dead viability dye panel) developed to enter only dead cells. Secondly, we selected CB, a commercially available fluorescent dye used to investigate membrane permeability.

We found that in OATP1B or 2B1 expressing live insect cells a typical transporter mediated uptake of ZV can be observed (Fig. 1). Moreover, a robust OATP-mediated ZV uptake was also confirmed in A431 cells engineered to overexpress the OATP transporters (Figs 2 and 3). ZV was designed to label cells with compromised membranes. Indeed, in control Sf9 and A431 cells ZV fluorescence correlated with propidium iodide staining (Fig. 1a and Supplementary Figure 5). However, our results demonstrate that the expression of OATPs results in the staining of living (propidium iodide negative) cells, warranting caution in the interpretation of results obtained with ZV as a viability dye.

Using the Sf9 expression system we identified another viability dye, Live/Dead Green as an OATP1B and 2B1 substrate (Fig. 1c). However, further large scale screens required a well-adherent cell line with stable OATP expression. Therefore we generated A431 cells with OATP1B1, 1B3 or 2B1 overexpression. Although A431 is not routinely used in pharmacological applications, due to its well-adherence it is well suited to microplate based assays^43^. Unexpectedly, only very low levels of OATP1B1 and 1B3 expression could be achieved in this cell line (Fig. 2a) and also in HEK293 or MDCKII cells. Enrichment of OATP1B expressing living cells based on antibody labelling is not feasible due to the lack of an anti-OATP antibody recognizing an extracellular epitope. Therefore we sorted cells based on increased fluorescence associated with OATP1B/2B1mediated LDG uptake, which led to the enrichment of OATP1B and 2B1 positive cells characterized by elevated OATP expression and function (Fig. 2c,d). The application of a fluorescent dye to enrich OATP-expressing cells is a unique (and to our knowledge the first) tool that allows the generation of cells with high OATP levels.

To validate the reliability of the A431 model, we also tested the uptake of the newly identified fluorescent substrates in HEK293 and MDCKII cells that are routinely used in transporter interaction studies. Results obtained in HEK and MDCKII cells overexpressing OATP1B1, 1B3 or 2B1 (Supplementary Figure 6) were fully consistent with those obtained in A431 cells, supporting the conclusion that the novel fluorescent dye substrates can be applied to characterize OATP1B/2B1 function.

Consistently with studies showing that an acidic extracellular milieu can significantly stimulate OATP-mediated transport^35,42^, we observed transport of the new fluorescent substrates almost exclusively at pH 6.5–5.5. Whether this is due to chemical changes in the fluorescent molecules at lower pH or an indication of proton counter transport, needs further investigation. Importantly, inhibition constants obtained with the novel assays at acidic pH are in full harmony with data obtained at neutral pH^34,41,44^, indicating that the established assay conditions are suited for OATP drug interaction screens.

Although the exact transport kinetics (K_m_ and V_max_ values) for ZV, LDV and LDG could not be defined (the molecular formula of these dyes could not be obtained from the suppliers due to proprietary concerns), we were able to determine and compare transport kinetics of CB and AF405 with that of Fl-MTX. Fl-MTX is a well-established substrate of OATP1B1 and 1B3^32^, and we have demonstrated previously that Fl-MTX is also transported by OATP2B1 when an acidic extracellular environment is generated^36^. In A431 cells we could confirm the OATP2B1-mediated uptake of Fl-MTX, however compared to OATP1Bs, Fl-MTX was found to be a poor substrate of OATP2B1 (Figs 2 and 3). In the case of the novel dye substrates, we found that CB is preferentially transported by OATP1B1 and 2B1 as compared to Fl-MTX, while OATP1B3 shows preferential transport of AF405 as compared to CB and Fl-MTX (Figs 3 and 4 and Table 2).

Besides the hepatic OATP1Bs and 2B1, OATP1A2 is also an important drug transporter. However, in our pilot experiments, we found no detectable transport of Zombie Violet, Cascade Blue or Alexa Fluor 405 by this transporter.

Highly fluorescent dyes with low cell permeability and elevated transport by OATPs are ideal candidates for the development of a sensitive functional assay. Our results demonstrate that the novel OATP1B and 2B1 substrates and the established A431 model cells can be used to measure OATP function in a low to medium throughput format. The z-factor for the novel substrates also suggests that ZV, LDV (1B1, 2B1), LDG, CB (1B1, 2B1) and AF405 may be applied in large scale drug screening studies (Table 2). Experiments using CB or AF405 as probe substrates demonstrate that these dyes are suitable to detect OATP substrate/inhibitor interactions (Fig. 5). The IC_50_ values obtained in our assay are in good agreement with those measured with widely accepted test substrates (estrone-3-sulphate or estradiol-glucuronide) (Table 3). It is well known that OATP1Bs and 2B1 have more than one substrate binding site. The IC_50_ values measured for OATP1B1-mediated CB uptake are in harmony with those obtained with dichlorofluorescein and tritiated estradiol-glucuronide^34,44,45^. Therefore the CB assay may be a good alternative to test OATP1B1 drug interactions as a substitute to estradiol-glucuronide. Similarly, the AF405 and CB assay for OATP1B3 and 2B1, respectively resulted in IC_50_ values similar to that obtained in assays using tritiated estradiol-glucuronide (1B3)^44^, or estrone-3-sulphate (2B1)^44,46^ as probe substrates. These results again clearly argue that CB and AF405 are good alternatives to these radioactive assays. Interestingly, BSP at low concentrations activated CB or AF405 uptake by OATP2B1 or 1B3, respectively. Such an activation by BSP has not yet been documented, however the stimulatory effect of one compound to the transport of another is a well-known phenomenon (summarized in^47^). In the case of OATP1B3, progesterone was shown to stimulate Fl-MTX^32^ and epigallocatechin gallate estrone-3-sulphate uptake^48^. Progesterone has also an activating effect on OATP2B1-mediated estrone-3-sulphate and dehydroepiandrosterone sulphate uptake^49^, and prostaglandin A1, testosterone and fendilin on estrone-3-sulphate uptake^44,50^. One possible explanation may be a co-transport of the two molecules, however reciprocal transport has not yet been confirmed in any of these cases, and also needs further investigation for the fluorescent dyes and BSP.

In conclusion, we show here that several fluorescent viability dyes and two sulfopyrenes (CB and AF405) are high capacity substrates of the multispecific OATP1B and 2B1 transporters. The fluorescence-based transport assay measuring the uptake of the best-performing substrates, CB and AF405 open the way to the development of sensitive high-throughput assays for the detection of OATP1B/2B1 drug interactions.

## Materials and Methods

### Materials

Zombie dyes (Violet, Green) were purchased from BioLegend^®^ (San Diego, CA, US). LIVE/DEAD^®^ Fixable Cell Stain Dye panel, Cascade Blue hydrazide, Alexa Fluor 405 succinimidyl ester were bought from Thermo Fischer Scientific (Waltham, MA, US), and fluorescein-methotrexate triammonium salt from Biotium (Hayward, CA, US). Restriction endonucleases were from New England Biolabs Ltd. (Ipswitch, MA, US). All other materials, if not indicated otherwise, were purchased from Sigma Aldrich, Merck (Budapest, HU).

### Generation of plasmid constructs

Generation of baculovirus vectors (pAcUW21-L/OATP and pAcUW21-control) was described earlier^36^. OATP2B1 expressing cells were generated by transposase mediated genomic insertion of the OATP2B1 cDNA (BC041095.1, HsCD00378878). Briefly, OATP2B1 cDNA was amplified (Phusion1 High-Fidelity PCR Kit, NEB, Ipswitch, MA, US) from the vector obtained from Harvard PlasmID Repository (Harvard Medical School, Boston, MA, US) by using the following primers: forward 5′: GTAAAT GCGGCCGC AA GAATTC GCCACCATGGG ACCCAGGATAGG and reverse 5′ GTACAT GCGGCCGC T AAGCTT TCACACTCGGGAATCCTC. The PCR fragment was cloned between the NotI-HindIII sites of the pSB-CMV vector^51^.

OATP1B1 and 1B3 overexpressing cells were generated by lentivirus transduction. The lentivirus based pRRL-CMV-MCS-IRES-ΔCD4 vector was generated by replacing the sequence of GFP with a multicloning site of the pRRLSIN.cPPT.PGK-GFP.WPRE (Addgene #12252) plasmid (Didier Trono, Lausanne, Switzerland). An IRES was cloned between the PmlI and XbaI sites of the MCS (forward: 5′-ACACGTGTCCGGACTAGTCCACCTTGCCTTACACATGAAGAG, reverse: 5′-ATCTAGAATGATCAGCCATATTATCATCGTGTTTTTCAAAG). The plasmid also contains a truncated CD4 receptor enabling monitoring of the virus transfection. Truncated CD4 cDNA was PCR amplified by the following primers (based on^52^: 5′-GATTCTAGAGCCACCATGAACCGGGGAGTCCCTTTTAGGC and 5′-GTAGTCGACTTAGCGCCTTCGGTGCCGGCAC from the pCMV-SPORT6-CD4 (Harvard Plasmid Repository). After digestion with XbaI-SalI enzymes, the PCR fragment was cloned to the corresponding sites of the pRRL-CMV-MCS-IRES vector.

The open reading frames OATP1B1 (Gene ID: AB026257) and OATP1B3 (BC141525, HsCD00348132) were amplified by HF PCR (Phusion1 High-Fidelity PCR Kit, NEB, Ipswitch, MA, US) from the pAcUW-21-L/OATP1B1-wt vector and from the plasmid obtained from Harvad PlasmID, respectively, using the following primers:

OATP1B1: forward 5′ TATTATTCGAAGCCACCATGGACCAAAATCAACAT, reverse 5′ CATGTAACTAGTTTAACAATGTGTTTCACTATCT.

OATP1B3: forward 5′ ACTAGTTTAAACGCCACCATGGACCAACATCAACAT and reverse 5′ GTACATGCGGCCGCACTGCAGTTAGTTGGCAGCAGCATTGTC. After digestion with BstBI and SpeI (OATP1B1) or PmeI-PstI (OATP1B3) enzymes the PCR fragments were cloned to the corresponding sites of the pRRL-CMV-MCS-IRES-ΔCD4 vector.

The base order of the cDNAs in the final vector constructs was verified by sequencing. Empty vectors without the OATP cDNAs, pSB-CMV and pRRLdCD4 were used as negative controls (indicated on the Figures as mock).

### Expression in insect cells

Transient expression of human OATP1B1, 1B3 and 2B1 in Sf9 (Spodoptera frugiperda) cells was achieved as described earlier^36^. For transport measurements, Sf9 cells after 36–40 hours post infection were used.

### Generation of cell lines

A431 cells (ATCC) were transfected with 1 µg plasmid DNA (OATP2B1) + 100 ng plasmid containing the transposase^51,53^ using Fugene HD reagent (Promega, Madison, WI, US) according to the protocol of the supplier. Puromycin (1 µg/ml) selection was started 48 h later. After 2 weeks of puromycin selection the cells were grown in DMEM (Gibco, Thermo Fischer Scientific (Waltham, MA, US)) supplemented with 10% fetal calf serum, 2 mM L-glutamine, 100 U/ml penicillin, and 100 μg/ml streptomycin at 37 °C with 5% CO_2_ and 95% humidity, without puromycin.

OATP1B1 and 1B3 overexpression in A431 cells was achieved by recombinant lentiviruses as described in^54^. HEK 293 T human embryonic kidney cells (1.8 × 10^6^ cells on a Petri dish (6 cm in diameter)) were transfected with (6 µg) pRRL-CMV-MCS-IRES-ΔCD4/OATP1B1 or OATP1B3, 2.2 µg pMDG and 4 µg psPax2 vectors^54^ using CaPO_4_ precipitation. The supernatant, containing lentiviral particles was collected 72 h after the transfection. Transduction of target A431 cells was carried out on 6 well plates. The multiplicity of infection was approximately 1.

### Determination of dye uptake

#### Flow cytometry

In order to determine the uptake of the fluorescent molecules in Sf9 cells, recombinant baculovirus infected cells were collected 36–40 hours post infection. After washing in the appropriate buffer (usually uptake buffer pH 5.5, see below) 5 × 10^5^ cells were incubated at 37 °C with the appropriate amount of dyes (the exact concentrations/amounts are indicated in the Figure legends) in a final volume of 100 µl. Transport experiments were carried out in the uptake buffer (125 mM NaCl, 4.8 mM KCl, 1.2 mM CaCl_2_, 1.2 mM KH_2_PO_4_, 12 mM MgSO_4_, 25 mM MES, and 5.6 mM glucose, with the pH adjusted to 8.5, 7.4, 6.5 or 5.5 using 10 N NaOH or 1 M HEPES). Incubation time was between 1–60 minutes. The reaction was stopped by the addition of 1 ml ice-cold phosphate-buffered saline (PBS). The cells were kept on ice until flow cytometry analysis. The cellular fluorescence of min. 20,000 live cells was determined using an Attune Acoustic Focusing Cytometer (Applied Biosystems, Life Technologies, Carlsbad, CA, US). Dead cells labelled with propidium iodide (PI, 1 µg/ml) were excluded. Functional data for each OATP represent the mean of at least 3 independent experiments performed on different days. In the case of A431 cells, cells were collected after trypsinization (0.1% trypsin) and the uptake experiments were performed in the same way as described for insect cells (see above). Data presented on Figures were generated by the FCS Express software.

#### Microplate-based assay

For the microplate-based assay, OATP-expressing A431 cells were seeded (6 × 10^4^ cells in 200 µl final volume/well) onto 96-well plates and cultured for 16–24 h at 37 °C, 5% CO_2_. Next day, the supernatant was removed and the cells were washed 3-times with 200 µl of PBS. When inhibitors were tested, the cells were pre-incubated in the presence of inhibitors (solved in DMSO) for 5 min at 37 °C (usually in 50 µl volume). The amount of DMSO was kept below 0.5% throughout the study. This amount of the solvent did not influence the fluorescence of the dyes. The reaction was started with the addition of 50 µl fluorescent dye (1–40 µM final concentration or 0.05 µl–1.2 µl in final volume of 100 µl) and the plate was incubated at 37 °C for 2–30 minutes. The reaction was stopped by the addition of 200 µl ice-cold PBS. The supernatant was rapidly removed, and the cells were washed 3-times with 200 µl ice-cold PBS. Finally, 200 µl PBS was added to the cells and fluorescence was measured at room temperature using an Enspire fluorescent plate reader (Perkin Elmer) at wavelengths indicated in Table 1.

#### Cell sorting

Function-based sorting was carried out based on the Live/Dead Green uptake of A431 cells expressing OATP1B1, 1B3 or 2B1. 2–4 × 10^6^ cells were incubated with 0.8–1.2 µl Live/Dead Green in 100 µl of transport buffer (sterile filtered), pH 5.5 at 37 °C for 30 minutes. The reaction was stopped by the addition of 1 ml DMEM and the cells were centrifuged at 300 g for 4 minutes. The cell pellet was suspended in 500 µl DMEM. Cellular fluorescence was analysed using a BD FACSAria III Cell sorter (BD Biosciences, San Jose, CA, US). Cells with the highest fluorescence (see the applied gate (“LDG+”) on Fig. 2) were collected and cultured for further analysis. Cells kept in culture for maximum 20 passages were used for the experiments.

#### Western blot

Whole cell lysates of Sf9 or A431 cells (10–50 µg) were separated on 7.5% Laemmli SDS-PAGE gels and transferred onto PVDF membranes. Immunoblotting was performed as described in^36^. Membranes were incubated overnight with OATP-specific antibodies or anti-β-actin antibody (A1978, Sigma). The antibodies used for the detection of OATP1B1 and 2B1 were kind gifts from Dr. Bruno Stieger (Department of Clinical Pharmacology and Toxicology, University Hospital, 8091 Zurich, Switzerland)^55^. The antibody raised against OATP1B3 (AMAb91231) was purchased from Atlas Antibodies (Stockholm, Sweden). Secondary antibodies used were 10,000–20,000x diluted, HRP-conjugated anti-rabbit or anti-mouse antibodies (Jackson ImmunoResearch, Suffolk, UK). Luminescence was detected using the Luminor Enhancer Solution kit by Thermo Scientific (Waltham, MA, US).

#### Toxicity measurements

5 × 10^3^ A431 cells were seeded onto 96-well plates in a final volume of 100 µl DMEM. The next day a transport assay was performed at sterile conditions using 0.4 or 1.6 µl Live/Dead Green or Zombie Violet, respectively/5 × 10^5^ cells in a 100 µl final volume. After 30 minutes, the cells were washed twice with PBS. Finally, 200 µl DMEM was added and the cells were cultured for 144 hours. Viability of the cells was determined using the PrestoBlue (Thermo Fischer Scientific) assay. Briefly, the medium was removed, and 100 µl 5% PrestoBlue in PBS was added to the cells. After incubation for 60 minutes at 37 °C, absorbance was detected at 583 nm with an Enspire fluorimeter (Perkin Elmer). Cells incubated with the buffer alone served as control. Background signal was calculated by absorbance measured in empty wells filled with 5% PrestoBlue.

### Data analysis and statistics

Z-factor was calculated as follows: 1 − ((3 × SD_negative control_ + 3 × SD_positive control_)/(Mean_positive_ − Mean _negative_)) based on^37^. Kinetic parameters of dye uptake or inhibition were analysed by Hill fit using the Origin 8.6 software. Statistical significance was calculated by Student’s t-test. The p value for statistical significance was set at 0.05 (*), 0.01 (**) or 0.001 (***).

### Data availability

The datasets generated during the current study are available from the corresponding author.

## Electronic supplementary material

Supplementary Information
