# Supplementary material for: Identification of novel cell-impermeant fluorescent substrates for testing the function and drug interaction of Organic Anion-Transporting Polypeptides, OATP1B1/1B3 and 2B1
Source: Sci Rep. 2018 Feb 8;8:2630. doi: 10.1038/s41598-018-20815-1 (PMC5805760; doi:10.1038/s41598-018-20815-1)
Supplement: Supplementary file 1 — Supplementary Information [file 41598_2018_20815_MOESM1_ESM.pdf]

Supplementary Figures

Identification of novel cell-impermeant fluorescent substrates for testing the function and drug interaction of Organic Anion-Transporting Polypeptides, OATP1B1/1B3 and 2B1

Izabel Patik, Virág Székely, Orsolya Német, Áron Szepesi, Nóra Kucsma, György Várady, Gergely Szakács, Éva Bakos and Csilla Özvegy-Laczka

Supplementary Figure 1

a

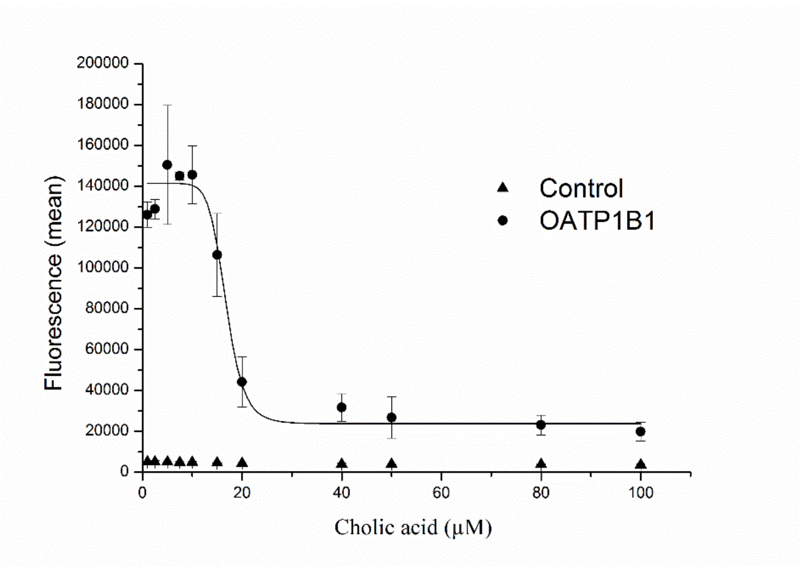

Supplementary Figure 1a. Inhibition of OATP1B1-mediated transport by cholic acid. Intracellular accumulation of 0.1 μl ZV / 5x10^5 cells in 100 μl was measured in the presence of increasing concentrations of cholic acid (0.1–100 μM) for 20 min. Data (means ± SD) obtained from 3 independent experiments are shown. Control stands for Sf9 cells overexpressing an unrelated protein, a Drosophila melanogaster telomerase subunit [1].

b

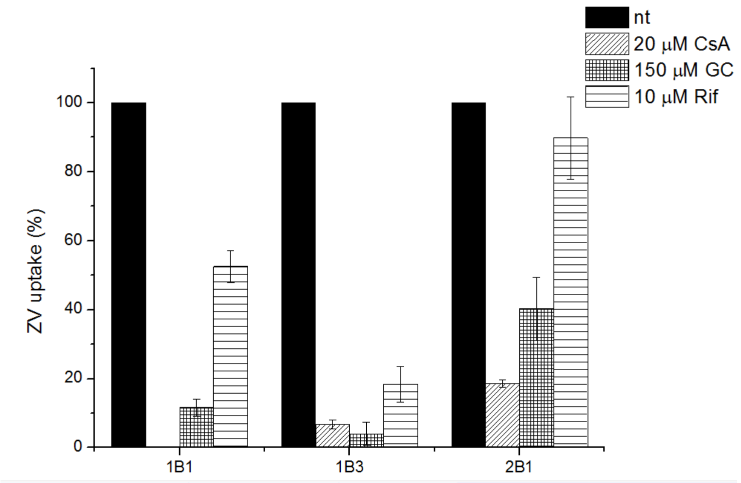

Supplementary Figure 1b. Inhibition of ZV uptake in Sf9 cells overexpressing human OATP1B1, 1B3 or 2B1. Intracellular accumulation of 0.1 μl ZV / 5x10^5 cells in 100 μl was measured in the presence or absence of inhibitors (CsA: cyclosporine A, GC: glycocholate, Rif: rifampicin) for 20 min. Data (means ± SD) obtained from 3 independent experiments are shown.

Supplementary Figure 2

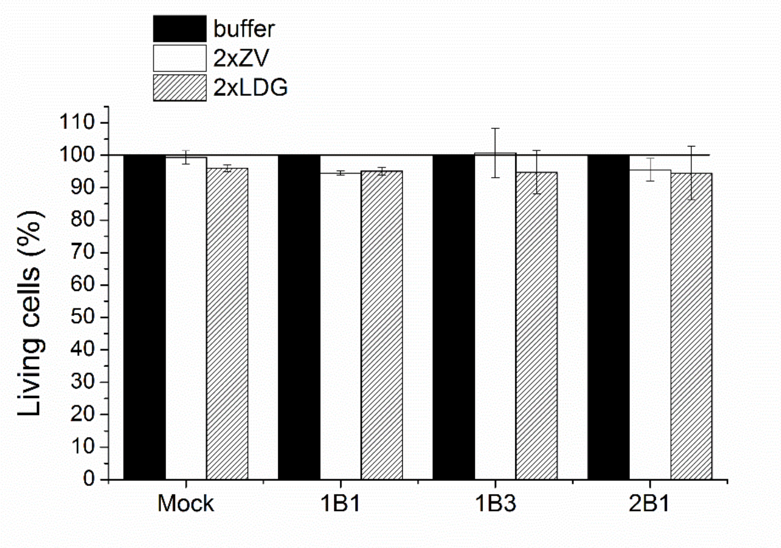

Supplementary Figure 2. ZV and LDG are non-toxic OATP substrates. A431-OATP cells were incubated with 1.6 µl ZV or LDG (in final volume of 100 µl, achieving 200% of the dye amount used for sorting) or empty buffer (uptake buffer pH 5.5) for 30 minutes. After washing, cells were grown for 4 days in DMEM. Viability of A431 cells was determined by the PrestoBlue assay. Absorbance measured in cells incubated in empty buffer was taken as 100%. The number of living cells in LDG or ZV treated cells was compared to this value. Average of 2 independent experiments ± SD values are shown.

Supplementary Figure 3

a

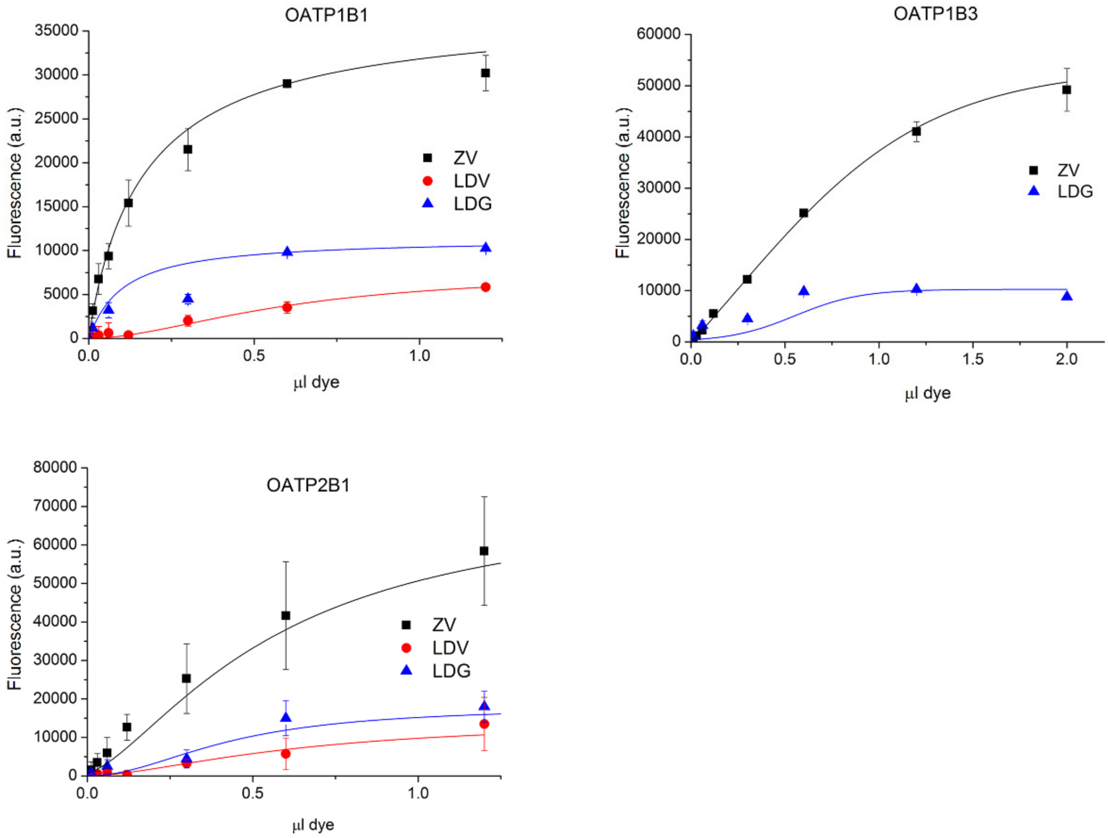

Supplementary Figure 3b

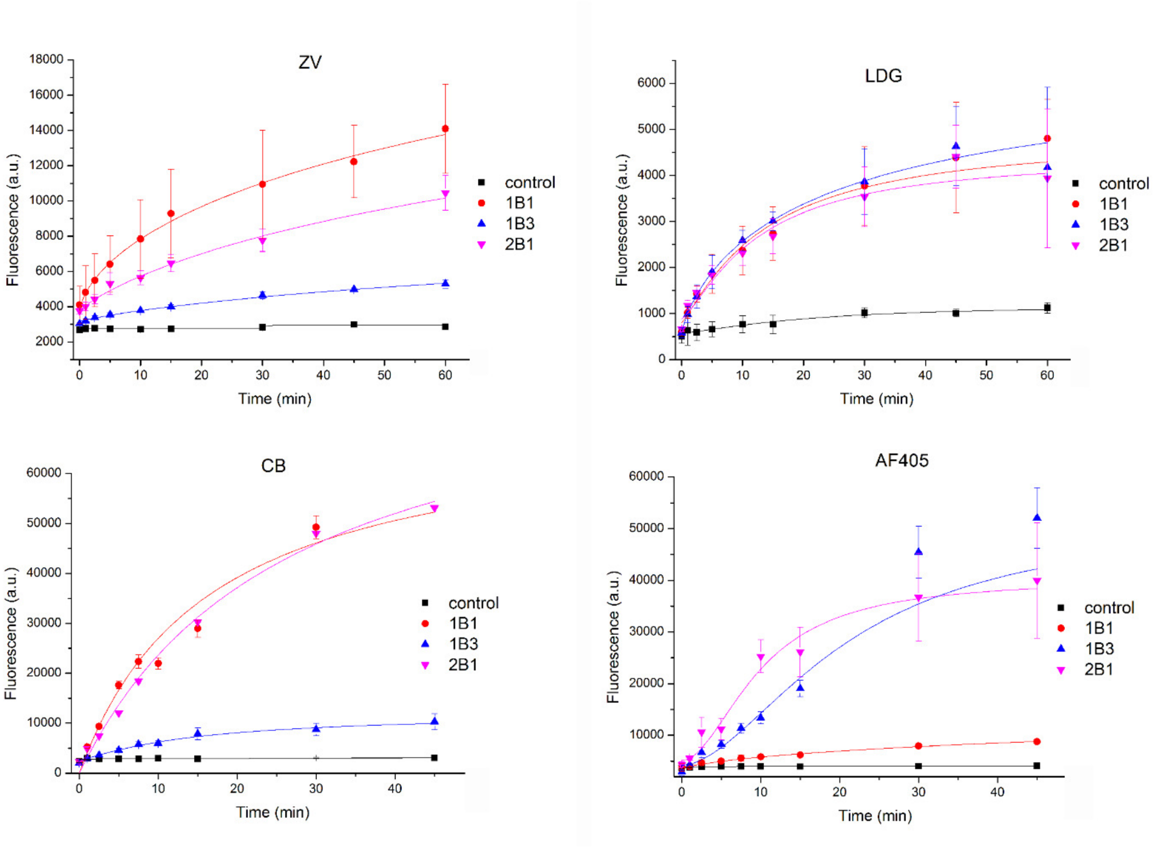

Supplementary Figure 3. Kinetics of dye uptake in A431 cells. Transport was measured in 96-well plates containing 1.2 x 10^5 cells/well. **a)** Cells were incubated with increasing amounts of ZV, LDG or LDV in the linear phase of uptake (10 min). Transport was determined by subtracting fluorescence in A431-mock cells. **b)** Cells were incubated with 0.05 µl (in final volume of 100 µl) ZV, 0.05 µl (in final volume of 100 µl) LDG, 10 µM CB or 10 µM AF405 for 60 minutes.

Kinetic parameters of dye uptake were analysed by Hill fit using the Origin 8.6 software. Experiments were performed at least in duplicates with three parallels in each biological replicate. Average ± SD values are shown.

Supplementary Figure 4

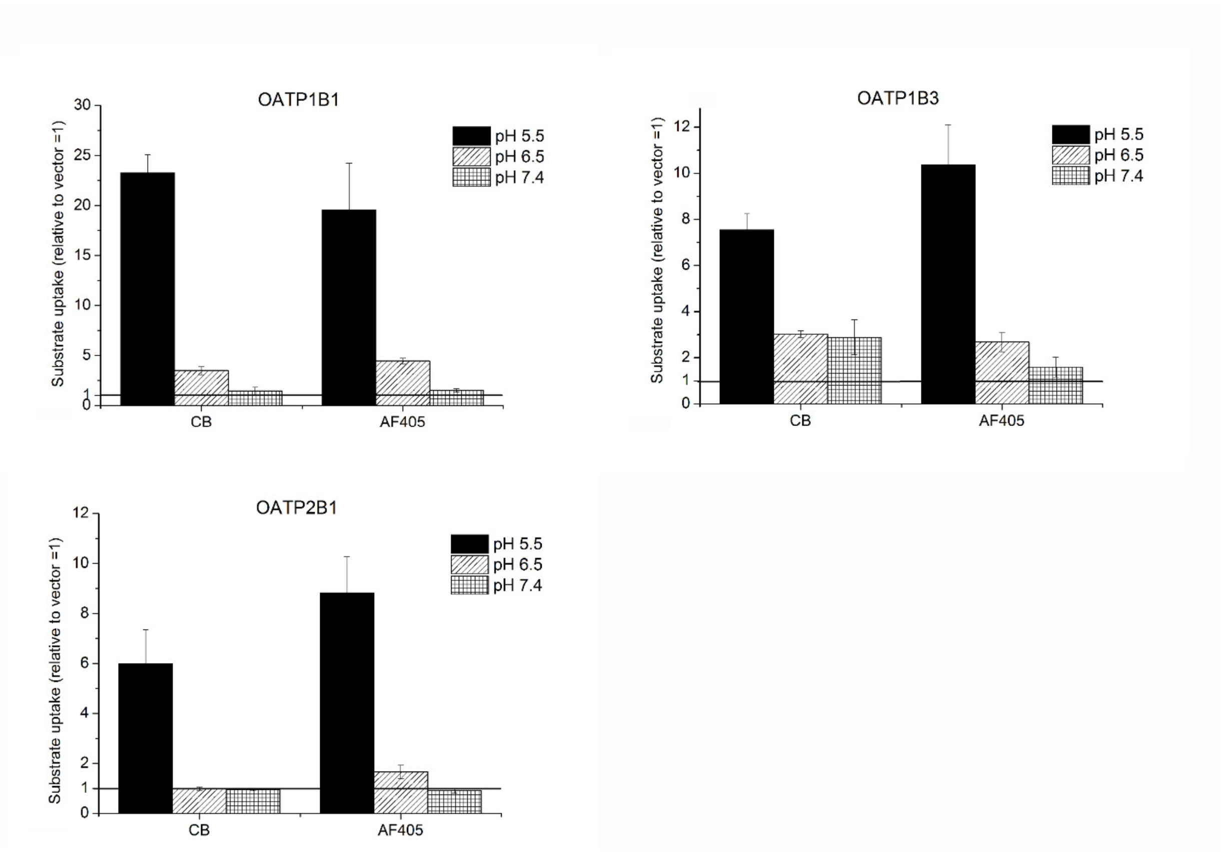

Supplementary Figure 4. pH dependence of dye uptake in A431 cells. Transport was measured in 96-well plates containing 1.2 x 10^5 cells/well. Cells were incubated with the dyes for 30 minutes in uptake buffer pH 5.5, 6.5 or 7.4. Dyes were added as follows: A431-OATP1B1 2 µM CB and 1 µM AF405; A431-OATP1B3 20 µM CB and 5 µM AF405; A431-OATP2B1 5 µM CB and 2.5 µM AF405. Experiments were performed in triplicates with three parallels in each biological replicate. Average ± SD values are shown.

Supplementary Figure 5

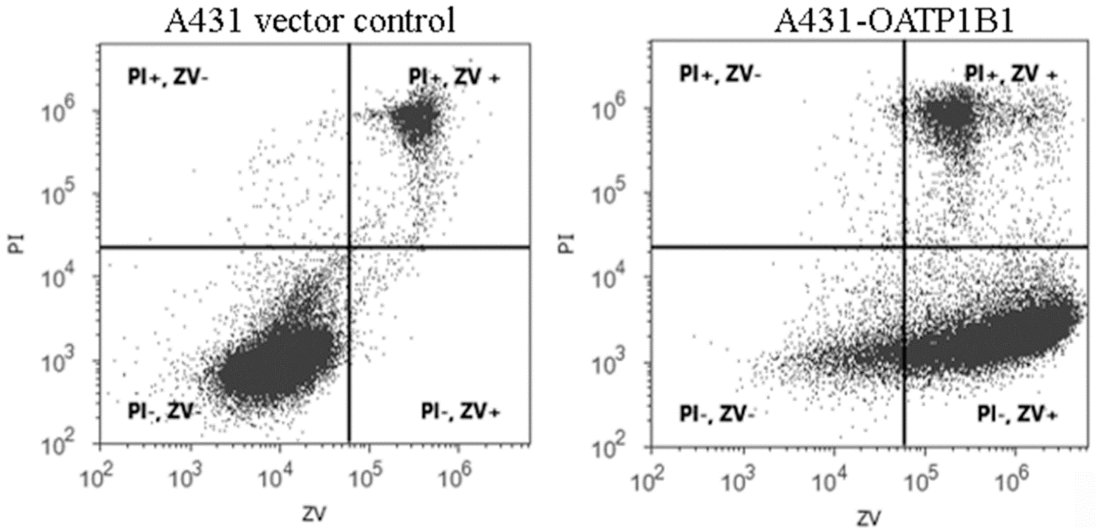

Supplementary Figure 5. Increased ZV uptake in live A431-OATP1B1 cells. Cells were incubated with 0.2 µl ZV (in final volume of 100 µl) at 37°C in pH 5.5 uptake buffer for 15 minutes. Fluorescence was measured by flow cytometry. Dead cells were identified based on PI (1 µg/ml) staining. Experiments were repeated at least three times, the result of one representative experiment is shown.

Supplementary Figure 6

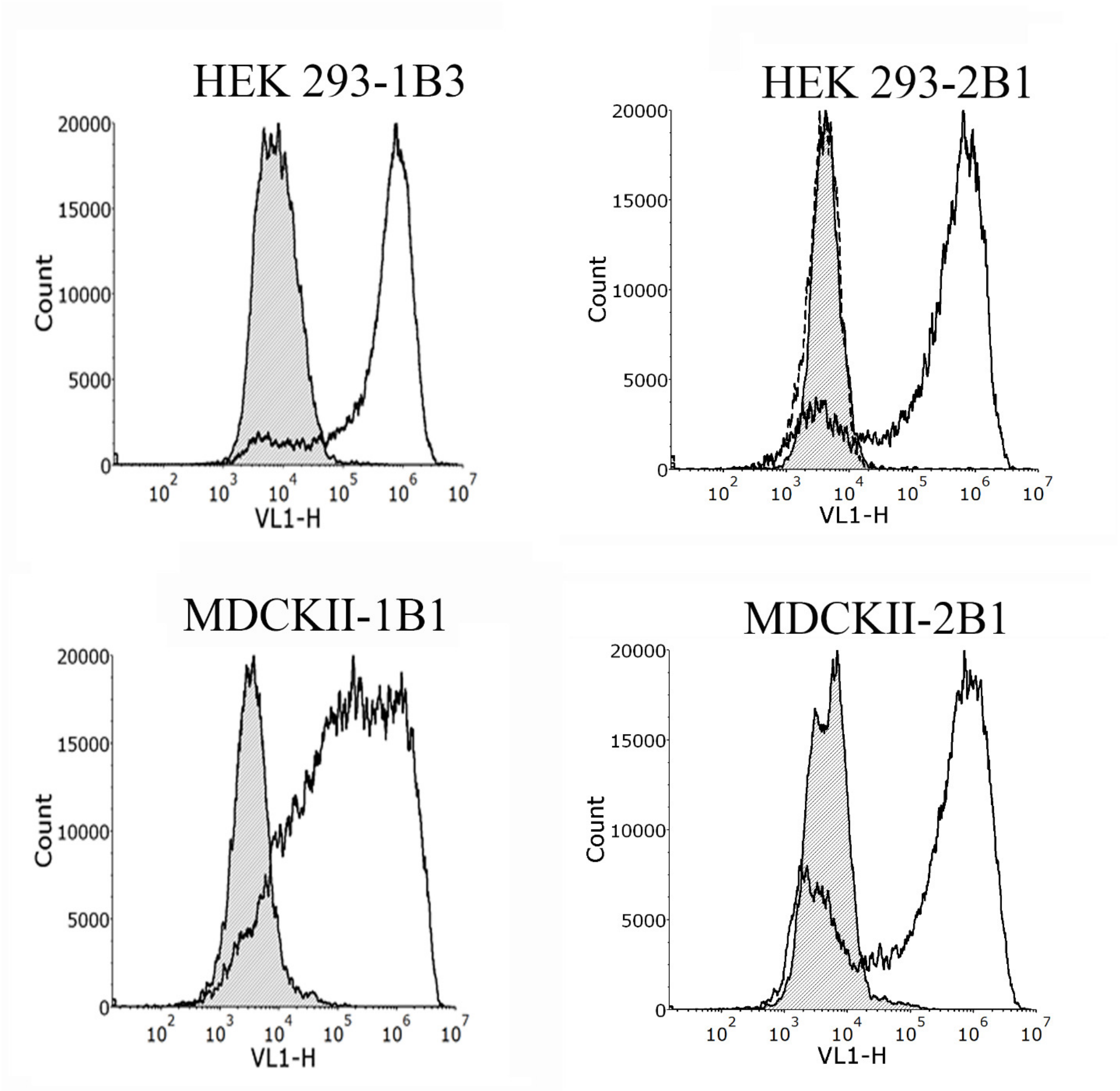

Supplementary Figure 6. Zombie Violet (ZV) uptake in HEK 293 and MDCKII cells expressing OATPs, 1B1, 1B3 or 2B1. Intracellular accumulation of 0.1 µl ZV / $5x10^5$ cells in 100 µl was measured at pH 5.5 for 30 min. Dead cells were excluded based on PI staining (1 µg/ml). Experiments were repeated at least two times. One representative experiment is shown. Filled histograms show fluorescence measured in cells transfected with the empty vector.

Supplementary Figure 7

a

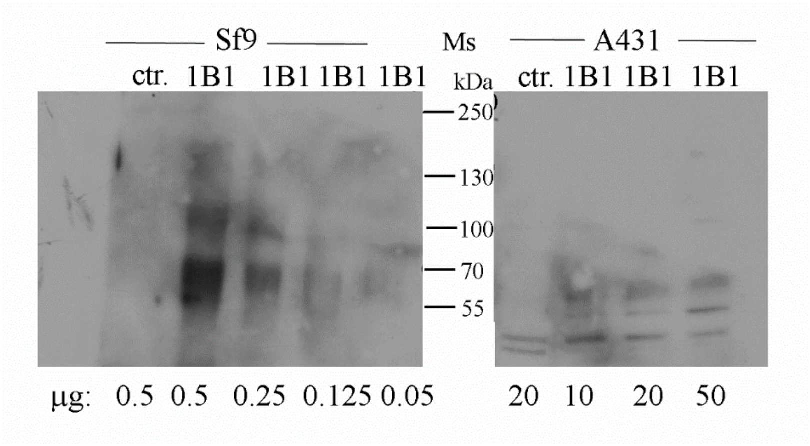

b

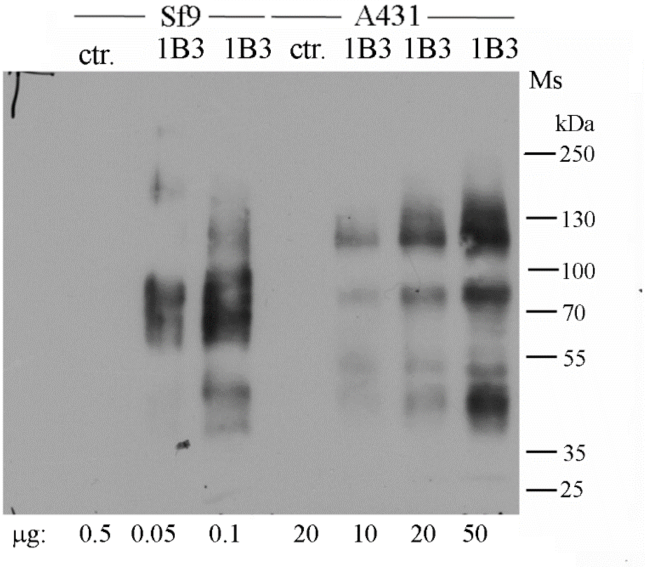

c

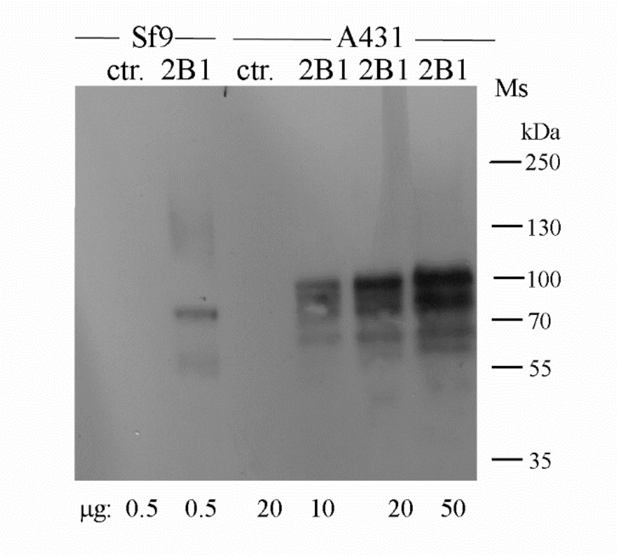
Sf9
A431
ctr. 2B1 ctr. 2B1 2B1 2B1
Ms
kDa
250
130
100
70
55
35
µg: 0.5 0.5 20 10 20 50

d

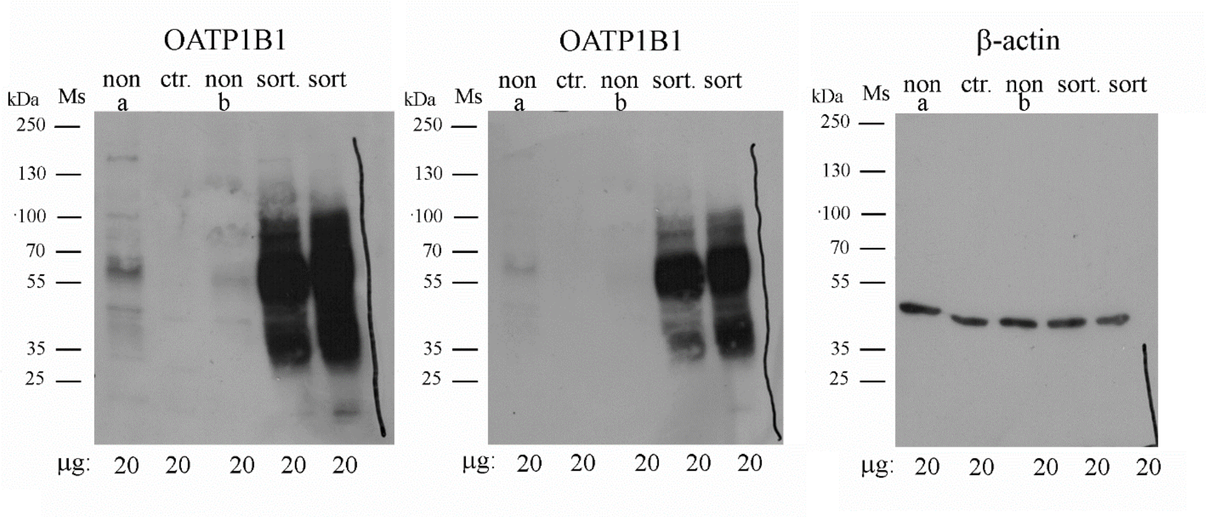
OATP1B1
OATP1B1
β-actin
kDa Ms
non a
ctr.
non b
sort.
sort
250
130
100
70
55
35
25
µg: 20 20 20 20 20

e

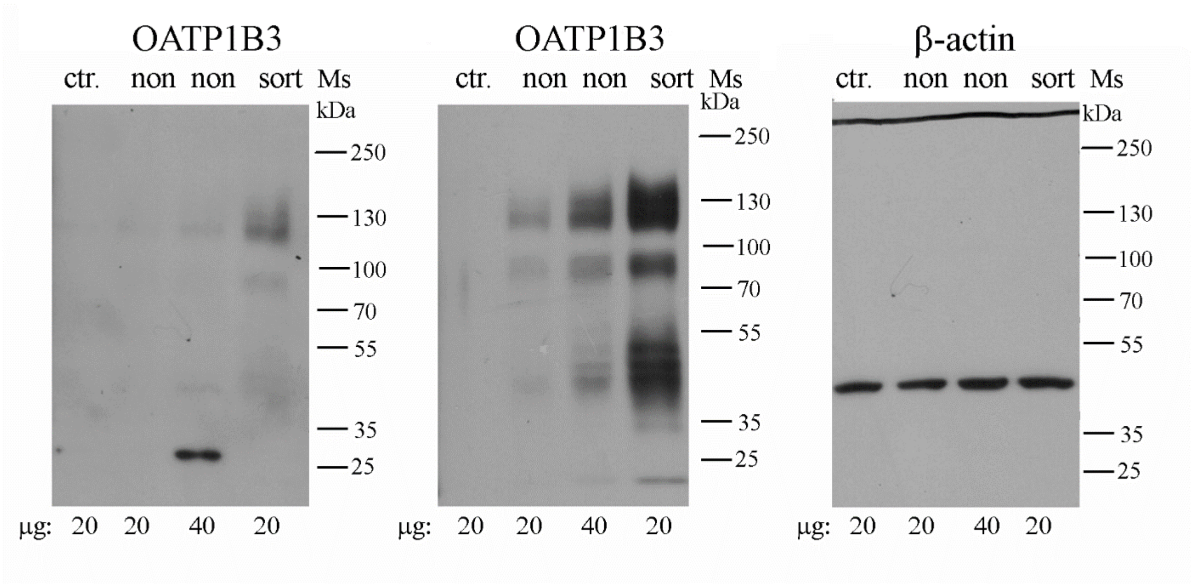
OATP1B3
ctr. non non sort Ms
kDa
250
130
100
70
55
35
25
µg: 20 20 40 20
OATP1B3
ctr. non non sort Ms
kDa
250
130
100
70
55
35
25
µg: 20 20 40 20
β-actin
ctr. non non sort Ms
kDa
250
130
100
70
55
35
25
µg: 20 20 40 20

f

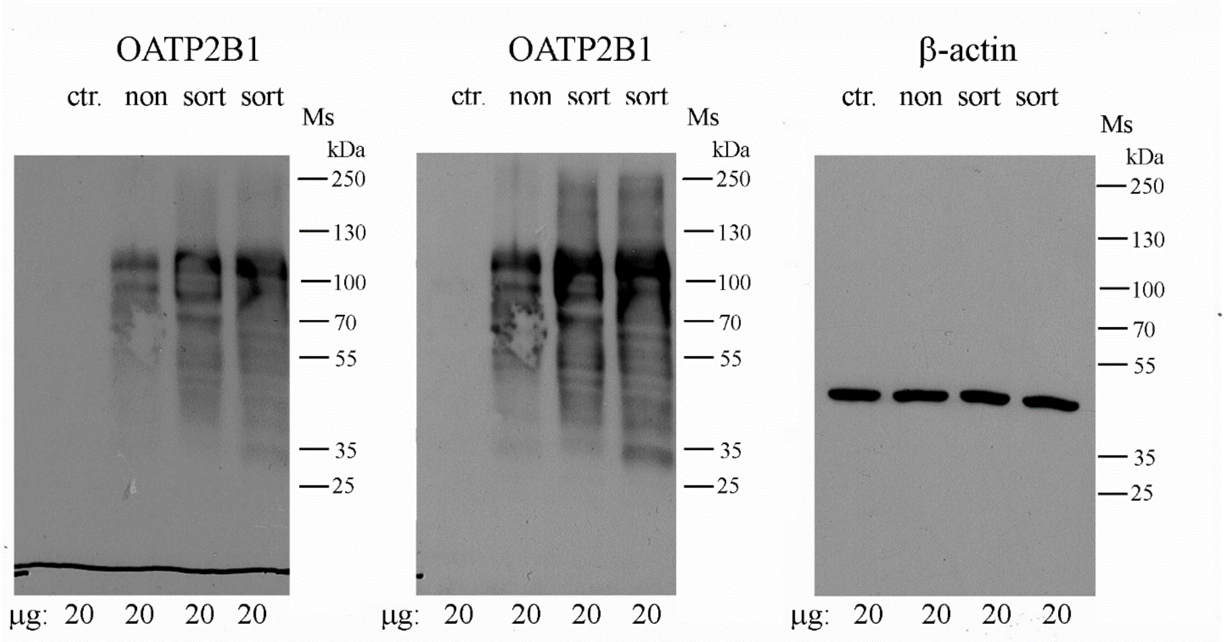
OATP2B1
ctr. non sort sort
Ms
kDa
250
130
100
70
55
35
25
µg: 20 20 20 20
OATP2B1
ctr. non sort sort
Ms
kDa
250
130
100
70
55
35
25
µg: 20 20 20 20
β-actin
ctr. non sort sort
Ms
kDa
250
130
100
70
55
35
25
µg: 20 20 20 20

Supplementary Figure 7. Original Western blots of Figures 2a and 2d. Total cell lysates were analysed by Western blot as described in the Methods section. Control (ctr.) represents Sf9 cells expressing an unrelated protein or mock transfected A431 cells. Multiple migratory bands most probably represent differently glycosylated forms of OATPs. "Ms" stands for PageRuler Plus Prestained Protein Ladder (Thermo Scientific, No. 26619) applied as a molecular weight marker.

a-c) Comparison of expression levels of OATPs achieved in Sf9 and A431 cells. a) Without sorting cells loose OATP1B1 expression. "non a" and "non b" samples were taken from the same cell line but "non b" was taken from cells after 4 weeks in culture.

d-f) Comparison of OATP expression in A431 cells before and after sorting. Two different exposures are shown. β-actin was used as loading control.

References:

1 Patik, I. *et al.* Functional expression of the 11 human Organic Anion Transporting Polypeptides in insect cells reveals that sodium fluorescein is a general OATP substrate. *Biochem Pharmacol* **98**, 649-658, doi:10.1016/j.bcp.2015.09.015 (2015).
